# Imogolite Nanotubes and Their Permanently Polarized Bifunctional Surfaces for Photocatalytic Hydrogen Production

**DOI:** 10.1002/gch2.202300255

**Published:** 2023-12-20

**Authors:** Erwan Paineau, Gilberto Teobaldi, Pablo Jiménez‐Calvo

**Affiliations:** ^1^ CNRS Laboratoire de Physique des Solides Université Paris‐Saclay Orsay 91405 France; ^2^ Scientific Computing Department STFC UKRI Rutherford Appleton Laboratory Harwell Campus Didcot OX11 0QX UK; ^3^ Chair of Thin Film Materials IZNF Friedrich‐Alexander‐ Universität Erlangen‐Nürnberg Cauerstraße 3 91058 Erlangen Germany

**Keywords:** band‐bending, hydrogen production, imogolite, photocatalysis, polarization

## Abstract

To date, imogolite nanotubes (INTs) have been primarily used for environmental applications such as dye and pollutant degradation. However, imogolite's well‐defined porous structure and distinctive electro‐optical properties have prompted interest in the system's potential for energy‐relevant chemical reactions. The imogolite structure leads to a permanent intrawall polarization arising from the presence of bifunctional surfaces at the inner and outer tube walls. Density functional theory simulations suggest such bifunctionality to encompass also spatially separated band edges. Altogether, these elements make INTs appealing candidates for facilitating chemical conversion reactions. Despite their potential, the exploitation of imogolite's features for photocatalysis is at its infancy, thence relatively unexplored. This perspective overviews the basic physical‐chemical and optoelectronical properties of imogolite nanotubes, emphasizing their role as wide bandgap insulator. Imogolite nanotubes have multifaceted properties that could lead to beneficial outcomes in energy‐related applications. This work illustrates two case studies demonstrating a step‐forward on photocatalytic hydrogen production achieved through atomic doping or metal co‐catalyst. INTs exhibit potential in energy conversion and storage, due to their ability to accommodate functions such as enhancing charge separation and influencing the chemical potentials of interacting species. Yet, tapping into potential for energy‐relevant application needs further experimental research, computational, and theoretical analysis.

## Graphical abstract

This Perspective concerns imogolite nanotubes and their multifaceted properties that can lead to beneficial outcomes in energy‐related applications. Their ease of nanostructuring combined with their permanent polarization and band‐bending features, present opportunities for novel design of advance photocatalytic strategies. This work illustrates two recent case studies demonstrating a step‐forward on photocatalytic hydrogen production achieved through chemical modifications.

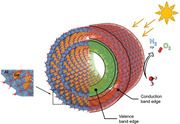

## 1 Introduction

The pressing need to attain carbon neutrality and zero emissions is driving the strong demand for sustainable chemical conversion and use of renewable green energy sources.^[^ ^1^, ^2^ ^]^ The goal is to reach 32% consumption by 2030 as established by the European Union (EU)à Energy Directive Council.^[^ ^3^ ^]^ Solar energy, holding terawatt potential, stands as a candidate in facilitating the energy transition.^[^ ^4^ ^]^ Notably, significant strides have been made in artificial photosynthesis, driven by photoelectrocatalytic contributions spanning the past two decades, especially in water splitting.^[^ ^5^, ^6^, ^7^, ^8^, ^9^, ^10^, ^11^ ^]^ As a result, the proliferation of visible‐light‐responsive high‐performance semiconductor materials—either bare or modified— has emerged as an appealing avenue.^[^ ^12^, ^13^, ^14^ ^]^ Furthermore, the exploration of conventional metal oxides, chalcogenides, carbon‐based materials, one‐dimensional (1D) nanoplatforms are typical, functional, and active catalysts.

Addressing the issue of photocatalysis involves processes taking place on short time scales but also on restricted distances.^[^ ^15^ ^]^ For the former, a variety of different time‐resolved spectroscopies is now employed for tracking picosecond to microsecond events in photocatalytic materials such as surface photovoltage, transient absorption, or time‐resolved spectroscopies (infrared, fluorescence, photoluminescence, microwave conductivity) to cite a few.^[^ ^15^, ^16^, ^17^, ^18^, ^19^, ^20^ ^]^ Scientists have also worked to understand how fundamental chemical and/or physical principles differ when photocatalytic systems are restricted to nanoscale dimensions.^[^ ^21^, ^22^, ^23^, ^24^ ^]^ The rise of nanoscience and nanotechnology has offered an ideal playground for exploring these concepts in a variety of different nanoreactors, from nanotubes to more complex three‐dimensional porous architectures.^[^ ^25^, ^26^ ^]^ The strong interest in nanotubes is due to their well‐defined, oriented porous structure combined with unique electro‐optical properties induced by the rolling up of the atomic lattices.^[^ ^27^ ^]^ Early on, these iconic nanoscale objects were recognized as ultimate 1D building blocks for next‐generation devices that could outperform current technologies for nanofluidic, molecular sieving, or energy storage.^[^ ^28^, ^29^, ^30^, ^31^ ^]^ In brief, the ion/energy storage capacity is primarily determined by the surface terminal groups,^[^ ^32^ ^]^ likely hydroxyls in the case of imogolite nanotubes (INTs). Furthermore, the hollow internal diameter enables ion hosting within the cavity, adding a secondary storage mechanism. As far as photocatalysis is concerned, literature is dominated by studies on TiO_2_ nanotubes^[^ ^33^, ^34^, ^35^ ^]^ although other photoactive metal‐oxide semiconductors nanotubes have been proposed.^[^ ^36^ ^]^ TiO_2_ nanotubes stand out as a noteworthy example.^[^ ^37^, ^38^ ^]^ One prominent TiO_2_ nanotubes platform exhibited competitive hydrogen production rates, ranging from 30 µmol h^−1^ g^−1^ to 80 mmol h^−1^ g^−1^.^[^ ^34^ ^]^ However, it's important to note that these rates were achieved in conjunction with the use of co‐catalysts. As for bulk materials, coupling nanotubes with metallic nanoparticles is usually necessary to promote the generation and separation of photoexcited carriers in the structure. Although the loading of noble metals as co‐catalysts is beyond the scope of this perspective, recent research findings highlight the potential of exploring this avenue as an alternative direction for improving hydrogen production rates.^[^ ^39^, ^40^ ^]^

Another strategy is to exploit the polarization effect that originates from the noncentrosymmetric arrangement of atoms in the structure.^[^ ^41^, ^42^ ^]^ The permanent polarization induces positive and negative surfaces spatially separated into the catalyst, which may allow efficient electron‐hole separation. Although several photo‐active 2D and 3D porous nanostructures with permanent polarization have started to appear in the literature^[^ ^43^, ^44^ ^]^ the exploitation of 1D polarized nanotubes remains in its infancy. In the context, imogolite nanotubes emerge as a potentially interesting choice, given their prior use in environmental applications, ca. the degradation of dyes and pollutants.^[^ ^45^, ^46^, ^47^ ^]^ Notably, INTs’ cross‐sectional separation of the band edges and permanent polarization may be exploited for more elaborate chemical conversion reactions, specifically in energy applications such as hydrogen evolution reaction (HER).^[^ ^48^ ^]^

In initial section, this perspective emphasizes the primary features and physical‐chemical properties of INTs, defining their potential significance as a innovative wide bandgap insulator. In the following section delves two case studies that exemplify groundbreaking outcomes in H_2_ production achieved through cutting‐edge material modifications: atomic doping and Schottky junction interfacing. Lastly, opportunities, directions, and modifications are proposed to continue unleashing INT's potential.

## 2 Main Features and Physical‐Chemical Properties

### 2.1 Bifunctional Surface: Inner and Outer of the Tube

**Figure 1:**
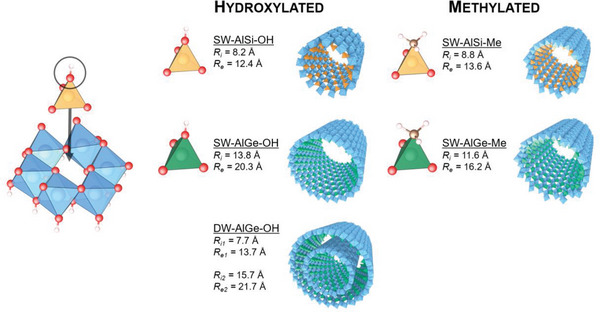
(Left) Atomic structure of the dioctahedral aluminum layer (blue) and of the location of an SiO_4_ tetrahedron (orange). Oxygen atoms are in red and hydrogen ones are white. (Right) Representation of the different members of the whole family of imogolite nanotubes with hydroxylated or methylated inner cavity. Orange tetrahedral correspond to aluminosilicate imogolite nanotubes (INTs) while green tetrahedral are aluminogermanate INTs. Inner (outer) radius of the nanotubes is provided. Adapted from ref.[76]

Imogolite nanotubes are unique 1D nanostructures, first recognized in weathered volcanic soils as true hollow tubes,^[^ ^49^ ^]^ 30 years before the emergence of the “nanotube era.”^[^ ^50^ ^]^ The chemical composition of INTs is defined as (OH)_3_Al_2_O_3_Si(OH) from outside to inside the tube wall. It consists of an outer di‐octahedral Al(OH)_3_ wall on which isolated tetrahedral of O_3_Si(OH) are covalently bonded upright to the octahedral vacancy, forming the inner surface of the nanotube wall (**Figure** **1**). The advantage of INTs in contrast to other nanotubes arises from a well‐defined minimum of strain energy,^[^ ^51^ ^]^ which leads to a defined number of tetrahedra along the nanotube circumference.^[^ ^52^ ^]^

There are no imogolite deposits to speak of, and their extraction from soils requires several purification operations before a sufficient quantity can be obtained. On the other hand, synthetic INTs can be obtained by straightforward methods using low‐temperature hydrothermal protocols.^[^ ^53^, ^54^ ^]^ The benefit is the ability to control or modify the nanotube crystallochemistry by changing the synthesis conditions and obtain new imogolite‐like (or geo‐inspired) nanostructures.^[^ ^55^, ^56^ ^]^ One of the most striking examples is the replacement of Si atoms by Ge ones,^[^ ^57^ ^]^ which allows the direct synthesis of monodisperse nanotubes in diameter with adjustable pore size (1–3 nm), shape (single‐walled: SW; or double‐walled: DW) and nanotube length through changes in the substitution rate (Si_x_Ge_1‐x_) or the synthesis procedure (Figure 1).^[^ ^58^, ^59^, ^60^, ^61^, ^62^, ^63^ ^]^ These nanotubes all have the same chirality with a zig‐zag configuration.^[^ ^52^, ^64^, ^65^ ^]^

The peculiar structure of INTs with inner and outer hydroxyl groups also makes them excellent candidates for surface modification. It has been widely documented that the outer wall can be functionalized with various coupling agents such as noble‐ and/or transition metals^[^ ^66^, ^67^, ^68^ ^]^ and polymers.^[^ ^55^, ^69^ ^]^ More interestingly, the inner cavity of hydrophilic INTs can be rendered hydrophobic by a postsynthesis chemical modification. Postfunctionalization can be obtained by different silane agents after a perfect dehydration of the hydrophilic INT samples, but the degree of inner surface substitution is lower than 35%.^[^ ^70^ ^]^ A more convenient way is the direct, template‐free, synthesis route by replacing, for instance, the initial alkoxide (prefiguring the tetrahedral layer) by a functionalized one.^[^ ^71^, ^72^, ^73^ ^]^ Like for hydrophilic INTs, a dependence of the inner cavity diameter with the substitution rate is obtained from the silicon to the germanium analogue end‐members with a progressive increase of the nanotube diameter.^[^ ^74^ ^]^ Interestingly, methylated‐INTs roll up into an armchair structure^[^ ^75^ ^]^ unlike their zig‐zag hydroxylated analogs, which may strongly impacted their optical and electronic properties. Overall, synthetic INTs offers a whole family of 1D nanoporous structures with functionalized interfaces, namely single ‐walled aluminosilicate (SW‐AlSi‐OH), aluminogermanate (SW‐AlGe‐OH), methylated aluminosilicate (SW‐AlSi‐Me), and methylated aluminogermanate (SW‐AlGe‐Me) nanotubes as well as double‐walled aluminogermanate (DW‐AlGe‐OH) nanotubes (Figure 1).^[^ ^76^ ^]^

### 2.2 Permanent Polarization

**Figure 2:**
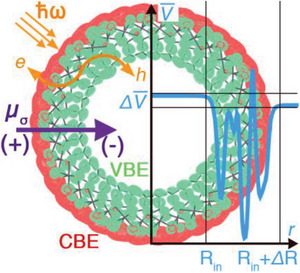
Schematic representation of the permanent dipole density (*µσ*) at the wall of imogolite nanotubes, and associated interface electrostatic potential step (Δ*V*), that can be used to polarize differently reactants inside and outside the NT cavity, offering potential control of their electron‐transfer kinetics. The panel shows also the *µσ*‐induced real‐space separation between the valence band edge (VBE) (green) and conduction band edge (CBE) (red), and intra‐wall charge‐transfer excitations (wiggly arrow) separating electrons (e^−^) and holes (h^+^) on different sides of the NT‐cavity. Picture from Ref. [G4]. Reproduced with permission.^[^ ^80^ ^]^ Copyright 2023, Wiley.

Owing to their tubular geometry and compositional radial symmetry, the INTs invariably present a permanent dipole density across the single or double wall interface. This feature, first inferred from electrophoresis measurements by Gustaffson in 2001,^[^ ^77^ ^]^ has been directly^[^ ^78^, ^79^, ^80^, ^81^ ^]^ or indirectly^[^ ^82^ ^]^ confirmed by density functional theory (DFT) as well as tight‐binding DFT (TB‐DFT)^[^ ^83^ ^]^ simulations of several members of the imogolite family. Regardless of the zigzag or armchair rolling, the INTs present, at DFT level, accumulation of negative and positive charge on the inner and outer surface, respectively. One notable exception is provided by the results for the SW‐AlGe‐Me nanotubes, which presents an inverted dipole density with the negative and positive ends pointing toward the outer and inner surface, respectively.^[^ ^75^ ^]^ As shown in **Figure** **2**, the dipole density across the nanotube walls is accompanied by a marked separation of the valence band edge (VBE) and conduction band edge (CBE) on opposite sides of the nanotube cavity, which has been suggested to be conducive to the presence of long‐range charge‐transfer optical excitations.^[^ ^79^, ^80^, ^84^ ^]^ General, composition agnostic electrostatic models describing the interplay between the permanent dipole density and associated electrostatic potential step, thence polarizing electrostatic field (vide infra) across the nanotube wall(s) have been provided in a prior study.^[^ ^80^ ^]^ Preliminary simulations indicate such a permanent polarization to be partially resilient to the presence of point‐defects, which introduce very local perturbations in the nanotube electrostatics.^[^ ^78^, ^84^ ^]^ Notably, recent simulations of highly idealized, thence inevitably approximated, structures for the tube ends^[^ ^81^ ^]^ suggest the INTs can develop, in addition to a radial dipole density, also a longitudinal polarization and ensuing band bending due to physical truncation and ensuing structural relaxation.^[^ ^85^ ^]^

### 2.3 Band‐Bending of Adsorption and Desorption Species

DFT simulation of different hydroxylated and methylated INTs indicates that the dipole density across the wall, and associated electrostatic potential steps (Figure 2), is effective in tuning not only the adsorption geometry and energies of interacting molecules such as H_2_O but also the energy alignment of the molecules’ electron acceptor and donor levels.^[^ ^86^ ^]^ Combined with the demonstrated effects of the nanotubes structure and electrostatic environment in altering the molecular motions at the nanotube–H_2_O interfaces,^[^ ^87^, ^88^ ^]^ these findings prompt for additional research in the potential of INTs and associated radial polarization for the design and control of electron transfer kinetics at H_2_O interfaces as necessary for improved, up‐scalable photocatalytic strategies to hydrogen generation.

The possibility of introducing optical absorption in the visible range of the spectrum by heteroatom doping leading to potentially beneficial photocatalytic pathways not present in the pristine nanotubes has motivated growing computational and experimental research in the subject.^[^ ^46^, ^89^, ^90^, ^91^, ^92^, ^93^ ^]^

The nanotube polarization will self‐evidently polarize differently different molecular species (with different polarizability). Suggestions have accordingly been put forward also on the potentially beneficial use of INTs as co‐photocatalyst, i.e., as support for grafted molecular or nanoparticle‐based photocatalysts.^[^ ^79^ ^]^ As the polarizability, i.e., response to the electric field from the nanotube dipole density will be invariably different for H_2_O molecules and the grafted photocatalysts, novel and favorable band alignments may/might be engineered and realized, in principle even using systems of known unsuitability for water photoreduction (e.g., Fe_2_O_3_ and WO_3_)^[^ ^94^ ^]^ due to their insufficiently high CBE with respect to the H_2_/H_2_O redox potential.

## 3 Benefit Summary of Imogolite Nanotubes as Photoelectrocatalysts

The distinctive combination of bifunctional surface, permanent polarization, and band‐bending modulation brings specific properties in alumino(germano)silicate nanotubes, enabling new chemical potentials for driving the typical energetic reactions, namely H_2_O splitting, CO_2_ reduction, and N_2_ fixation. The adaptability of nanostructuring single or double walls becomes particularly interesting for improved photon management. Unlike conventional semiconductors, INTs demonstrate the ability to transport photogenerated carriers across their walls. As a result, these multifaceted nanotubes hold the potential to manipulate the chemical potentials of model photocatalytic reactions with minimal material modifications.

Together the polarization, surface, and band‐bending of INTs may enable unusual but distinctive effects with promising performance benefits:

– Increase the charge carrier's concentration and continuous flux after photon activation.
– Enhancement of carriers’ migration to the exposed outer surface since the distance length between the single and double walls are relatively short.
– Improvement on time scale of adsorption and desorption species by polarization and surface charge affinity with reactant molecules.
– Downward and upward of chemical potentials of model photocatalytic reactions when modified.

## 4 Hydrogen Production upon Photocatalytic and Radiolytic Activation

Two case studies were selected from two French teams with extensive experience in INT modifications over a decade. These teams have started to explore their catalytic performance using different activation sources. The discussion of this section centers on appreciating the unique aspects of each study, given the substantial differences in experimental conditions, rather than directly comparing the material's modifications nor performance. Particularly, regarding the activation sources (nature: photon or radiation, lamps: monochromatic, full spectrum, and power intensities). Nevertheless, each catalytic case study plays a role in positioning imogolite's in the energy applications (HER) and both are carefully analyzed to reveal its potential as nano‐reactors.

**Figure 3:**
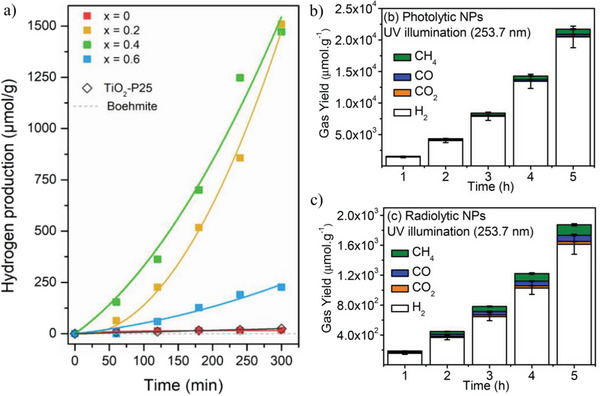
a) Photocatalytic hydrogen production of Ti‐modified Imogolite NTs under Xenon lamp illumination,^[^ ^95^ ^]^ b) photolytic, and c) radiolytic hydrogen production of imogolite nanotubes (INTs) functionalized with −CH_3_ groups and containing Au NPs.^[^ ^96^ ^]^

Firstly, Jiménez et al. developed Ti‐modified aluminogermanate double‐wall imogolite nanotubes.^[^ ^95^ ^]^ The authors reported 1500 µmol^−1^ g^−1^ HER (**Figure** **3**a) for the optimal 0.4 Ti/Ge‐INT composite with typical sacrificial agent concentration (methanol 33% volume) and full Xe lamp (300 W) irradiation.^[^ ^95^ ^]^ Interestingly, the present result was obtained without any co‐catalyst thus positioning in the metal‐free category. The high photoactivity was attributed to low recombination rates and accumulated electrons at the available Ti‐sites, which presumably present longer lifetimes.

Rather than relying on metal nanoparticles as co‐catalysts and electron collectors, the hybrid active composite's inherent polarization facilitates enhanced photogenerated charge separation. Methanol decomposition functions to block the oxygen evolution reaction (OER) while generating protons simultaneously. These processes occur within the bifunctional surface nanotube, leading to effective spatial separation of useful charge carriers, with proton reduction taking place in the outer wall of the NT.

Secondly, Patra et al. reported a value of 1443 µmol^−1^ g^−1^ HER (Figure 3b) in the case of aluminosilicate INT‐CH_3_ containing 20% Au NPs as co‐catalyst with propan‐2‐ol 20% as sacrificial agent and monochromatic light (254 nm, 15 W) irradiation.^[^ ^96^ ^]^ Notably, such INT‐CH_3_/Au photoactive composite exhibits an efficient metal‐semiconductor interface, leading to a substantial gold catalytic surface contribution, while the possibility of polarization and confinement effects from the INT‐CH_3_ cannot be disregarded.

INT‐CH_3_/Au composite has proven another interesting trend in function of size‐dependent. Patra et al. reported the comparison of 5 and 10 nm average size of Au NPs given a pronounced difference on hydrogen production of 10 and 90 times higher than bare INT‐CH_3_/Au. This outcome indicates the presence of metallic Au and highlights the active site on the outer surface of INT‐CH_3_. This site effectively converts nearby protons into H_2_. The second source used was gamma radiation from ^137^Cs in argon atmosphere (Figure 3c). However, the H_2_ production was one order of magnitude lower than when photoactivated, suggesting that the reduction mechanism via radiation does not bring the same benefits as photon to create a feasible metal‐semiconductor Schottky barrier.

## 5 Conclusions and Outlook

The chosen case studies on H_2_ generation via photocatalytic, photolytic, and radiolytic highlight the experimental proof‐of‐concept, evidence the clear potential of INTs as a strong candidate to be added to the list of topical semiconductor photoelectrocatalysts. Extensive progress has been achieved in characterizing diverse aspects such as physico‐chemical, optoelectronic, magnetic, bulk, and surface properties of this nanotube. However, it is crucial to include an examination of charge carrier dynamics in order to complete the comprehensive understanding of its behavior.

Considering the two presented case studies, it is evident that INT holds great potential to be further developed into a widely in‐demand metal oxide with remarkable properties, not just for energy conversion but also for energy storage. Although the latter aspect was not covered in this article, interested readers can find comprehensive details elsewhere.^[^ ^30^ ^]^ On the other hand, the introduction of imogolite double‐wall nanotubes provides a novel 1D nanostructure with controlled photon management for activation, opening up potentially exciting avenues for new research and advances in hydrogen/oxygen generation, carbon dioxide reduction, and nitrogen fixation, all of which guarantee further investigations.

On the horizon, there are additional opportunities for incorporating metal dopants, coupling with medium band gap semiconductors, and/or depositing bimetallic catalysts to continue harnessing and exceeding the current efficiencies of solar fuels technologies.

To further and better support future research efforts in the potential of INT for photo‐catalytic applications, two lines of simulation development would be very beneficial. First, definition and validation of scalable yet sufficiently accurate routes to modelling realistic electronic ground state interfaces between the nanotube's surfaces, interacting molecules and surrounding media has remained challenging over the last 10 to 15 years. Current advances in DFT‐derived machine learning interatomic potentials hold great promise to this end. Second, viable simulation methods for optical electronic excitations in the nanotubes beyond the single‐particle picture used so far as well as first principles approaches to excited state (excitonic) dynamics in model Imogolite‐water (solvent) nanotube interfaces are currently missing or yet to be applied. While the continuous increase in academically accessible high performance computing resources authorizes outlooks toward the use of many‐body approaches to valence‐electron optical spectroscopy, progress on the latter front will require intense method development efforts. At least to best of our knowledge, the needed computational machinery is yet to be developed or made available.

As for electrocatalytic applications, it is recommended to measure/report the INTs conductivity and/or resistivity. One can think of implementing significant vacancies or atoms in their lattice that can enhance their conductivity, and therefore electron collection for pure electrocatalysis.

## Conflict of Interest

The authors declare no conflict of interest.
